# A violência oculta na sola do chinelo: punição corporal, violência intrafamiliar e os modelos de autoridade em São Paulo, Brasil

**DOI:** 10.1590/0102-311XPT110324

**Published:** 2025-04-28

**Authors:** Renan Theodoro

**Affiliations:** 1 Universidade de São Paulo, São Paulo, Brasil.

**Keywords:** Maus-Tratos, Poder Familiar, Punição, Adolescente, Violência, Domestic Violence, Parenting, Punishment, Adolescent, Violence, Violencia Doméstica, Responsabilidad Parental, Castigo, Adolescente, Violencia

## Abstract

Violências intrafamiliares e castigos físicos são maus-tratos prevalentes no Brasil. Essas duas formas de maus-tratos são abordadas pela literatura de forma independente, com pouca ênfase na relação desses fenômenos com os modos como a autoridade familiar é exercida. Este estudo explora a relação entre violência intrafamiliar, punição corporal e autoridade familiar. Os dados são do *Estudo da Socialização Legal em São Paulo*, que, em 2016, entrevistou 800 crianças de 11 anos. As variáveis incluem regras da casa, consequências aplicadas pelo pais quando os participantes não cumprem as regras, percepção da justeza dos procedimentos adotados dos pais e convivência com violência intrafamiliar. Empregaram-se análise de correspondência múltipla e agrupamento hierárquico com base em componentes principais para identificar modos de autoridade familiar. Os resultados indicam três perfis: o *cluster* 1 representa relações não violentas e poucas regras impostas pela autoridade familiar, predominante em famílias de classe média; o *cluster* 2 caracteriza-se por punições não físicas e a adoção de procedimentos justos por parte das autoridades; o *cluster* 3 revela relações familiares violentas, com punições físicas e alta incidência de violência intrafamiliar, com representação significativa em famílias de baixa renda. Os resultados indicam que o castigo físico não é contingencial, mas uma prática comum em grupos familiares em que outras formas de violência intrafamiliar também estão presentes. Os achados contribuem para o refinamento de políticas públicas de enfrentamento à violência, bem como podem auxiliar profissionais na identificação de maus-tratos contra crianças e adolescentes.

## Abstract (en)

Intrafamily violence and physical punishment are prevalent forms of maltreatment in Brazil. These issues are often studied separately, with little emphasis on how they relate to the ways family authority is exercised. This study explores the relationship between intrafamily violence, physical punishment, and family authority. The data used come from the *São Paulo Study of Legal Socialization*, which interviewed 800 11-year-old children in 2016. Variables include household rules, parental responses to rule-breaking, children’s perception of fairness in parental discipline, and exposure to intrafamily violence. Multiple correspondence analysis and hierarchical clustering on principal components were used to identify patterns of family authority. The findings indicate three profiles: cluster 1 represents non-violent relationships with minimal imposed rules, predominant in middle-class families; cluster 2 is characterized by non-physical punishments and fair disciplinary practices; cluster 3 reveals violent family dynamics, with physical punishment and high levels of intra-family violence, prevalent in low-income families. The results suggest that physical punishment is not contingent, but rather a common practice in families in which other forms of violence are also present. These findings contribute to improving public policies to combat violence and can help professionals identify maltreatment against children and adolescents.

## Resumen

La violencia intrafamiliar y el castigo físico son maltratos frecuentes en Brasil. Estas dos formas de maltratos se han abordado en la literatura de forma independiente, con poco énfasis en la relación de estos fenómenos en la manera en que se ejerce la autoridad familiar. Este estudio explora la relación entre la violencia intrafamiliar, el castigo corporal y la autoridad familiar. Los datos se obtuvieron del *Estudio de Socialización Legal en São Paulo*, que había entrevistado a 800 niños de 11 años en 2016. Las variables incluyen las normas del hogar, las consecuencias aplicadas por los padres cuando los participantes no cumplían las normas, la percepción de la imparcialidad de los procedimientos adoptados por los padres y la convivencia con la violencia intrafamiliar. Se emplearon el análisis de correspondencia múltiple y la agrupación jerárquica basada en componentes principales para identificar los modos de autoridad familiar. Los resultados indican tres perfiles: El clúster 1 representa relaciones no violentas y pocas normas impuestas por la autoridad familiar, predominante en las familias de clase media; el clúster 2 corresponde a castigos no físicos y la adopción de procedimientos justos por parte de las autoridades; y el clúster 3 revela relaciones familiares violentas, con castigos físicos y una alta incidencia de violencia intrafamiliar, con una representación significativa en las familias de bajos ingresos. Los resultados indican que el castigo físico no es ocasional, sino una práctica común en grupos familiares en los que también están presentes otras formas de violencia intrafamiliar. Los hallazgos contribuyen al establecimiento de políticas públicas contra la violencia y proporcionan información a los profesionales para identificar situaciones de maltratos contra niños, niñas y adolescentes.

## Introdução

Os maus-tratos contra crianças e adolescentes são um problema global, presente em diferentes culturas e realidades socioeconômicas. A Organização Mundial da Saúde (OMS) estima que a cada ano mais de 40 mil crianças e adolescentes são mortos por violências ligadas a diversos tipos de maus-tratos, como violência física, sexual, psicológica e emocional. Além do risco à vida, os maus-tratos são uma grave violação de direitos humanos fundamentais, com consequências severas, e seus impactos podem ser sentidos até à vida adulta ^1^.

No Brasil, em 2024, completaram-se dez anos desde a aprovação da *Lei nº 13.010/2014* ^2^, legislação que visa lidar com maus-tratos praticados contra crianças e adolescentes, especialmente aqueles provocados por meio de castigos físicos. Trata-se de legislação que não prevê criminalização, e sua incidência se dá, principalmente, na promoção de políticas públicas de proteção a crianças e adolescentes. Em linhas gerais, a lei visa conscientizar a população quanto aos limites do exercício da violência física como forma pedagógica ^3^.

O principal desafio para a implementação desse tipo de lei é a falta de preparo dos profissionais que atuam nos mecanismos responsáveis pela identificação dos casos, sobretudo devido ao baixo conhecimento dos mecanismos de proteção ^4^. Também entre conselheiros tutelares podem ser notadas dificuldades para a identificação de violações contra crianças e adolescentes, especialmente quando se tratam de formas de violência que não deixam lesões aparentes ^5^.

Há outros indícios de que a sociedade é relutante em incorporar mudanças em relação aos maus-tratos contra crianças e adolescentes, e, ao que tudo indica, o castigo corporal ainda é amplamente aceito pela sociedade brasileira. Em pesquisa com amostra nacional ^6^, 39% das pessoas ouvidas afirmam que às vezes educar com castigos é melhor do que fazê-lo com diálogo, e 52% admitiram que já ameaçou bater como forma de educar ou corrigir o comportamento de uma criança.

Observando a produção acadêmica brasileira dos últimos dez anos, nota-se que os maus-tratos ora são tratados como violência intrafamiliar, ora como castigos corporais, e essas duas temáticas são abordadas frequentemente de forma independente. Além de indicar uma lacuna no conhecimento produzido sobre esses temas, essa dupla forma de abordar não contribui para a precisa identificação dos maus tratos pelas agências responsáveis.

Parte substancial dos estudos publicados sobre violência intrafamiliar procurou quantificar os casos a partir de fontes oficiais ou dados secundários. Por exemplo, Paungartner et al. ^7^ utilizaram dados do Sistema de Informação de Agravos de Notificação (SINAN) e identificaram 645.393 casos de violência contra pessoas de até 19 anos, entre 2009 e 2017. Mais de 60% das notificações identificadas eram das regiões Sul e Sudeste. Considerando apenas crianças e adolescentes na base do SINAN, Riba & Zioni ^8^ identificaram uma taxa de registros de violência doméstica da ordem de 187 casos por 100 mil habitantes, de 2009 a 2019. Ambos os estudos indicam que o principal local de ocorrência dessas notificações é a residência da vítima, e mães e pais figuram como os principais agressores ^7^ ^,^ ^8^.

A violência intrafamiliar reproduz desigualdades sociais marcantes do Brasil, especialmente aquelas ligadas ao gênero e à raça/cor das vítimas. As meninas prevalecem como vítimas na fase da adolescência, sendo que entre crianças predominam vítimas do sexo masculino ^7^. A partir de dados da *Pesquisa Nacional de Saúde do Escolar* (PeNSE), Antunes et al. ^9^ apontam que adolescentes negros apresentam maiores chances de relatar ter sofrido violência em casa.

Há menos dados disponíveis para traçar a magnitude nacional da punição corporal. Em compensação, os estudos publicados sobre esse tema permitem compreender qualitativamente as circunstâncias desses maus-tratos. A punição corporal ocorre sobretudo em decorrência das estratégias que pais, mães, responsáveis e cuidadores empregam para educar os filhos. Trata-se de uma forma de disciplinar e assegurar a obediência ^10^. Afeta mais frequentemente crianças do sexo masculino, e a mãe aparece com principal agressora.

Os fatores que explicam a violência intrafamiliar em geral também se aplicam ao caso da punição corporal: é mais provável que mães, pais e cuidadores que sofreram violências na infância apliquem castigos físicos, assim como a experiência de desemprego, o nível socioeconômico e a escolaridade das autoridades familiares estão associados à prática de punição corporal ^10^. Assim como outras formas de maus-tratos, a punição corporal pode ter como repercussão a reprodução da violência, com estudos indicando que adolescentes que sofrem punição corporal em casa podem apresentar comportamento agressivo na escola ^11^.

Na literatura internacional, a punição corporal tem sido abordada como parte das estratégias educativas de pais e responsáveis. As técnicas disciplinadoras se dividem em duas categorias: indutivas e coercitivas ^11^ ^,^ ^12^ ^,^ ^13^. As técnicas indutivas são aquelas que lidam com os comportamentos das crianças por meio do diálogo, incentivando que a criança compreenda as consequências de suas ações, estimulando a responsabilização; as técnicas coercitivas são aquelas em que se aplicam castigo físico e ameaças ^11^ ^,^ ^12^ ^,^ ^13^.

Outra forma de pensar essa distinção nas práticas de disciplina aplicada pelos pais/responsáveis e cuidadores é categorizá-las como “disciplinas construtivas” (como repreensão verbal, afastamento da criança temporariamente de redes sociais) ou “disciplinas não construtivas” (punição corporal, ameaça de punição corporal, xingamentos) ^14^ ^,^ ^15^.

Essas estratégias estão conectadas à noção de estilos parentais, às formas como os pais e responsáveis tentam regular o comportamento dos filhos e aos diferentes graus de apoio emocional. Nessa linha, pais e responsáveis podem ser categorizados de ao menos três formas: permissivos (muito flexíveis com regras e aceitando sem debate as demandas dos filhos); autoritários (muito rígidos nas regras, com baixo apoio emocional) e autoritativos (equilíbrio entre imposição de regras e apoio emocional) ^16^ ^,^ ^17^.

Outro aspecto fundamental da relação de autoridade é a justeza dos procedimentos adotados por pais e responsáveis ^17^. Apesar de o tema ainda ser incipiente no contexto brasileiro, estudos apontam que um elemento central para as relações de autoridade familiar é a avaliação que as crianças fazem a respeito dos procedimentos adotados pelos pais, sendo que autoridades que agem de forma justa, neutra, transparente e respeitosa são mais provavelmente vistas como legítimas ^14^ ^,^ ^15^.

Considerando essa breve revisão, a proposta deste artigo é explorar as associações entre violência intrafamiliar, punição corporal e exercício de autoridade familiar. Buscou-se a correspondência entre esses dois fenômenos, descrevendo as associações entre essas práticas, de forma que se possa compreender a punição corporal como extensão das violências intrafamiliares. O objetivo é identificar perfis de relação de autoridade familiar a partir de experiências de pré-adolescentes do Município de São Paulo com regras domésticas, consequências que enfrentam quando não obedecem às regras (incluindo a presença de punição corporal) e o convívio com violência intrafamiliar.

Essa forma de abordar o problema também deverá contribuir com a literatura por permitir comparar diferentes padrões de autoridade familiar, dando igual ênfase às formas não coercitivas de poder familiar. Ao destacar como os maus-tratos são parte das relações de autoridade familiar, e ao demonstrar outras formas possíveis de atuação desse tipo de relação social, espera-se contribuir para a melhor identificação e prevenção aos maus-tratos contra crianças e adolescentes.

## Métodos

### Fonte de dados

Os dados são do *Estudo da Socialização Legal em São Paulo* (SPLSS, sigla em inglês), um estudo desenvolvido pelo Núcleo de Estudos da Violência da Universidade de São Paulo. No ano de 2016, so estudo entrevistou 800 crianças matriculadas em escolas das redes pública e particular de ensino do Município de São Paulo. Estavam elegíveis a participar do estudo crianças nascidas no ano de 2005 e residentes do município. Dessa forma, os participantes tinham, em média, 11 anos de idade.

Primeiramente, gerou-se uma lista de escolas públicas e privadas do município, sorteando-se, em seguida, as que participariam do estudo pelo método de probabilidade proporcional ao tamanho (PPT), de forma que nenhuma das cinco regiões da cidade (central, norte, sul, oeste e leste) fosse sub-representada. As escolas sorteadas eram consultadas, e, uma vez que a pesquisa fosse autorizada, as equipes de campo visitavam as salas de aula de 6º ano, distribuindo Termos de Consentimento e Assentimento. Participavam da pesquisa apenas aquelas crianças cujos pais ou responsáveis expressaram autorização via termo de consentimento. Ao final, participaram do estudo 112 escolas, e 58,75% dos respondentes eram de escolas públicas. O estudo foi aprovado pelo Comitê de Ética da Universidade de São Paulo (parecer nº 1242582, CAAE 47171015.5.0000.5390).

A coleta de dados foi realizada pela empresa Ibope-Inteligência (https://www.ipec-inteligencia.com.br/). Os questionários tinham aplicação de aproximadamente 30 minutos, e as entrevistas face a face com as crianças foram administradas com auxílio do software Survey-To-Go (https://www.dooblo.net/). As entrevistas foram realizadas nos estabelecimentos de ensino durante o horário das aulas, em sala reservada pelas direções para tanto. As perguntas sobre renda mensal familiar foram coletadas de pais e responsáveis, por meio de um formulário que era encaminhado com os Termos de Consentimento.

### Variáveis

Regras da casa: lista composta por dez regras que os pais podem estabelecer para crianças em casa. A lista foi elaborada a partir de tarefas mais comuns que adolescentes do município apresentavam no estudo piloto do SPLSS ^18^. Consiste em questões ligadas à saúde (proibição de fumar), questões de ordem moral (proibir palavrões e exigir que fale sempre a verdade), bem como regras relativas aos cuidados com a casa e ao controle de horários. Para esta análise, os dez itens foram somados. Com base nessa distribuição e considerando a mediana dessas variáveis, os casos foram categorizados em: “poucas regras” (0 a 7), “médio” (8 regras) e “muitas regras” (de 9 a 10).

Consequências: os participantes foram questionados sobre as consequências de não cumprirem as regras da casa. As consequências variaram de chamadas de atenção a punições físicas. Uma das perguntas era se os pais “*chamavam atenção e davam bronca*”. Em estudos anteriores, essas questões foram categorizadas em “não violentas” e “violentas” ^14^ ^,^ ^15^. Neste estudo, cada consequência será analisada individualmente para uma melhor compreensão de sua associação com os diferentes perfis de autoridade.

Justeza dos procedimentos: participantes eram questionados “*se seus pais ou responsáveis achassem que você fez alguma coisa errada, eles...*”, e em seguida era apresentada uma lista, com os quatro aspectos da justeza dos procedimentos, conforme descrito por Tyler ^19^ e pelo campo da Socialização Legal ^20^: voz, transparência, imparcialidade e respeito. Devido à baixa variabilidade dos itens, as quatro dimensões foram somadas e, posteriormente, criou-se uma variável dicotômica. Assim, a variável está dividida entre indivíduos cujos pais não praticam nenhuma das dimensões (sem procedimentos justos = 19,87%) e aqueles cujos pais praticam todas as dimensões (com procedimentos justos = 80,12%).

Violência intrafamiliar: esta parte inclui perguntas a respeito de experiências com violência em casa. Perguntava-se aos participantes: “*Na sua casa, já aconteceu alguma dessas situações*” e apresentava-se a eles uma lista com três circunstâncias (brigas entre adultos, sofrer agressão física por um adulto e sofrer agressão física por outra criança ou adolescente). Suas respostas foram coletadas em uma escala Likert de frequência de quatro pontos (0 = nunca; 3 = muitas vezes). Para a análise, as respostas foram codificadas em três grupos: aqueles que não relataram vitimização em casa (44,5%); aqueles que relataram apenas uma vitimização em casa (45,12%); e aqueles relataram duas ou mais vitimizações em casa (10,37%).

Variáveis socioeconômicas: na análise de correspondência múltipla (ACM), as variáveis socioeconômicas são consideradas como “suplementares”, para não participarem nos cálculos das associações entre as variáveis de interesse ^21^. Foram incluídas como variáveis suplementares: (i) quantidade de irmãos; (ii) variável indicando se os participantes moram ou não com avós; (iii) religião; (iv) raça/cor (obtida pelo método de autoclassificação); (v) tipo de administração escolar; (vi) sexo; e (vii) renda familiar (em salários mínimos mensais), informada pelos pais ou responsáveis com o Termo de Consentimento.

### Análise

Para identificar os perfis de autoridade familiar, o estudo recorreu à ACM, seguida de agrupamento hierárquico com base em componentes principais (HCPC, sigla em inglês). Essas técnicas oferecem uma visão relacional de fenômenos sociais ^22^. Diferem de outras técnicas ao não isolar variáveis como “dependentes” ou “independentes” e ao não buscar efeitos de uma variável sobre outra, mas por buscar associações entre múltiplas variáveis simultaneamente ^23^.

Os resultados nesse tipo de análise são gráficos, como nuvens de indivíduos e distribuição das categorias das variáveis em planos cartesianos. A distância entre dois pontos do plano é calculada com base nas divergências das escolhas para as categorias das variáveis, de modo que indivíduos que selecionaram as mesmas categorias nas questões devem apresentar coordenadas similares ^24^. Dos 800 participantes do SPLSS, em 2016, 796 estavam com todas as respostas selecionadas completas, e apenas estes foram considerados, evitando, assim, técnicas de imputação.

## Resultados

Em relação às regras em casa, a maioria dos adolescentes mostra que seus pais exercem controle sobre questões de saúde e moral, como proibição de fumar e exigência de falar sempre a verdade. Outros temas, como controle dos amigos e horários, são menos frequentes, mas ainda comuns. A regra menos comum é sobre proibir que os filhos namorem. Considerando todas as regras, 25% da amostra apresenta até sete regras, 50% apresenta ao menos oito regras e 75% da amostra apresenta até nove regras.

Em relação às consequências aplicadas, as mais comuns entre os respondentes são os pais chamarem a atenção ou darem bronca (96,12%). Em razão dessa prevalência, este item não foi considerado na ACM, porque não permitiria diferenciar os indivíduos. A segunda consequência mais comum envolve castigos moderados ou a perda de algum direito, como assistir TV. São menos frequentes atitudes violentas, como xingar os filhos (15,37%) ou punição corporal, como bater (18,37%), embora 45,75% afirmaram que os pais ameaçam bater.

De maneira similar, a violência intrafamiliar atinge parcela restrita da amostra (4,65% afirmam ter sido agredido por chutes ou socos por um adulto, e 10,75% foram agredidos dessa forma por outra criança ou adolescente). No entanto, 51,68% presenciaram brigas ou discussões. Considerando todas as respostas, 44,5% dos participantes não sofreram nenhuma das três situações; 45,12% tiveram pelo menos uma experiência; outros 10,37% tiveram duas ou mais.

Para avaliar os resultados da ACM, o primeiro aspecto a ser considerado é o número de eixos que compõem o plano a ser analisado. A Tabela 1 indica que dois eixos explicam mais de 32,7% da variação dos dados. Caso incluíssemos três eixos, a explicação aumentaria para 43,7%, porém isso tornaria a análise dos planos mais complexa. Com base no princípio da parcimônia, optou-se por observar apenas dois eixos.

**Tabela 1:** Variância dos eixos/*eigenvalues*.

Eixo	*Eigenvalue*	Percentual de variância	Cumulativo
1	0,23	18,67	18,67
2	0,18	14,10	32,77
3	0,14	10,94	43,72
4	0,12	9,45	53,16
5	0,11	9,16	62,32

Considerando as contribuições de cada categoria para os eixos, o eixo 1 é principalmente influenciado pelas consequências aplicadas aos adolescentes quando violam as regras. Notavelmente, as consequências mais violentas são as que mais contribuem para a variação neste eixo (ter afirmado que os pais batem, gritam ou ameaçam bater). A categoria que mais influencia a variação no eixo 2 é a ausência de castigos aplicados pelos pais, seguida de “poucas regras”. Uma terceira categoria importante para compreender a variação ao longo do eixo 2 é aquela que indica ausência de procedimentos justos adotados pelos pais, de acordo com as percepções dos participantes.

**Figura 1:**
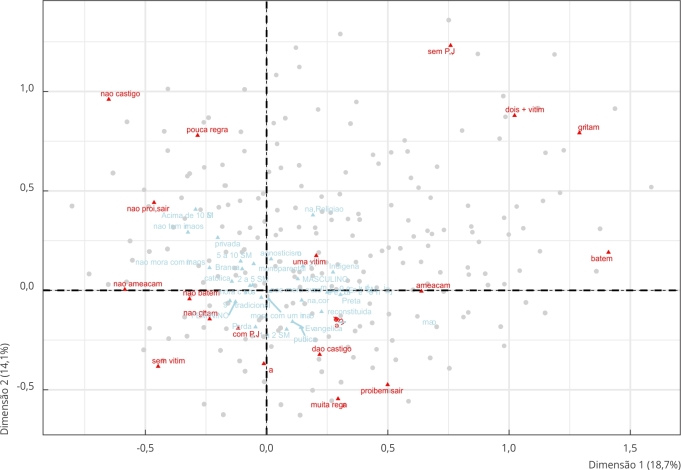
Mapa de indivíduos e categorias resultante da análise de correspondência múltipla.

A Figura 1 apresenta o mapa de indivíduos e categorias resultante da ACM. No primeiro quadrante (canto superior direito), encontraram-se participantes que afirmaram que seus pais e responsáveis adotam formas violentas de punição, como bater e gritar. Geralmente, essas crianças também estão expostas a mais de duas formas de vitimização, e a autoridade familiar é exercida sem consideração pelo que pensam os filhos, sem respeito, sem transparência (ausência de procedimentos justos).

Fonte: elaboração própria, com base em dados do *Estudo da Socialização Legal em São Paulo* (2016), realizado pelo Núcleo de Estudos da Violência, Universidade de São Paulo.

Ainda seguindo a Figura 1, no segundo quadrante (canto superior esquerdo), encontram-se participantes cujos pais ou responsáveis não impõem consequências, como punições, restrições ou ameaças de violência. No terceiro quadrante (canto inferior esquerdo), estão os adolescentes cujos pais evitam o uso da força e não aplicam consequências rigorosas quando ocorrem desobediências. Este grupo também inclui aqueles que não relatam experiências de vitimização em casa. Além disso, são os que mais acreditam na possibilidade de seus pais agirem de maneira justa e democrática. Por fim, no quarto quadrante estão os indivíduos que vivem em lares em que há um número médio de regras estabelecidas, castigos não físicos são aplicados e há ameaças de uso da força, embora não sejam efetivamente utilizadas.

Dois padrões são evidentes quando o plano é dividido verticalmente. No lado direito do eixo 1, encontram-se as práticas relacionadas à violência interpessoal. Indivíduos nessa área pertencem a famílias que usam violência ou ameaça de violência como método de punição com mais frequência do que aqueles no lado esquerdo. Pais que gritam com os filhos também estão mais associados a formas de punição que implicam violência física. Isso demonstra que a punição corporal está intimamente ligada a outras formas de violência intrafamiliar, além de apontar que dificilmente encontraríamos famílias que recorrem esporadicamente à punição corporal.

**Figura 2:**
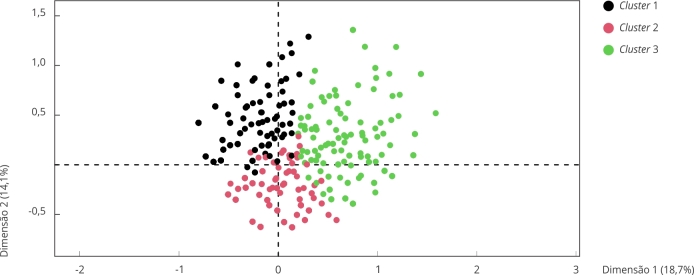
Mapa de indivíduos classificados por cluster.

A Figura 2 apresenta o mapa de indivíduos classificados por *cluster*, resultado da HCPC. Identificam-se três grupos diferentes. O *cluster* 1 agrupou 32,66% da amostra. É caracterizado por agrupar participantes que afirmaram que seus pais não batem, não gritam, não ameaçam bater nem colocam de castigo. É também um grupo cujos pais colocam poucas regras em casa. Do ponto de vista socioeconômico, é um grupo majoritariamente branco (57%), com estudantes matriculados na rede particular de ensino (56%). Além disso, 50% dos respondentes que não têm irmãos encontram-se neste *cluster*. Assim, pode-se afirmar que relações parentais não violentas e pouco estruturadas por regras é mais provável em famílias de classe média ou média alta, com um núcleo familiar pequeno.

Fonte: elaboração própria, com base em dados do *Estudo da Socialização Legal em São Paulo* (2016), realizado pelo Núcleo de Estudos da Violência, Universidade de São Paulo.

O *cluster* 2 reúne 46,18% dos respondentes. Este grupo é composto por indivíduos cujos pais impõem castigos, mas não de natureza física. Apesar de não ser comum o uso da força neste grupo, 44,98% dos membros deste *cluster* afirmam que os pais ameaçam bater. Assim como no *cluster* 1, a violência interpessoal em casa é relativamente rara para os membros do *cluster* 2, sendo que 57,18% dos participantes classificados neste grupo não relataram vitimizações em casa. Outro aspecto proeminente no *cluster* 2 é a presença de procedimentos justos por parte dos pais no exercício da autoridade, bem como apresenta uma quantidade significativa de regras estabelecidas pelos pais na dinâmica familiar. A maioria dos estudantes de escola pública foi classificada neste *cluster* (51,17%).

Finalmente, o *cluster* 3 representa 21,15% da amostra. É marcado por relações familiares mais violentas e punitivas. A maioria dos participantes deste cluster relata que seus pais gritam ou ofendem como forma de punição. Outra categoria distintiva deste grupo é a aplicação de punição física pelos pais, mencionada por 63,31% de todos os membros. Além disso, há uma alta incidência de violência intrafamiliar, com 65,06% dos participantes que sofreram duas ou mais vitimizações incluídas neste grupo. Notavelmente, o *cluster* 3 também se destaca pela baixa expectativa em relação à justiça dos procedimentos adotados pelos pais. Do ponto de vista socioeconômico, pré-adolescentes de famílias de até um salário mínimo estão mais representados neste *cluster*. Além disso, no *cluster* 3, estão participantes de famílias mais numerosas, com mais de um irmão.

## Discussão

Este artigo buscou compreender as circunstâncias em que ocorrem maus-tratos contra crianças em São Paulo, mais especificamente violências intrafamiliares e castigos corporais. O principal objetivo foi descrever modelos de autoridade familiar, explorando as maneiras como pais ou responsáveis exercem poder em situações cotidianas. Ao tratar o tema sob a perspectiva dos vínculos de autoridade, lembramos que o artigo possibilita a identificação das diversas formas que as relações familiares podem assumir, para além da prática violenta.

O estudo que ensejou o artigo apresenta limitações específicas. Os dados foram recolhidos com pré-adolescentes de 11 anos de idade no momento das entrevistas. As informações sobre vitimização foram informadas pelas próprias crianças, o que pode resultar em subnotificação ou notificação equivocada dos dados. Além disso, o instrumento de coleta não contava com um repertório extensivo de vitimizações. Essa limitação sugere a necessidade de pesquisas futuras que explorem a interconexão entre diferentes tipos de maus-tratos.

Os resultados revelam a existência de três modelos de autoridade familiar convivendo na realidade familiar no Município de São Paulo. Há o grupo de adolescentes que convivem com pais que aparentemente não estabelecem regras de comportamento, que são, a rigor, não punitivos e que agem como se a relação de autoridade estivesse ausente. No entanto, como aponta Richard Sennett ^25^, esse tipo de vínculo pode caracterizar-se mais pela dominação via indiferença do que pelo suposto respeito à autonomia, e a literatura também menciona as consequências que esse tipo de conduta pode gerar, como dificuldade futura por parte dos filhos em exercer o autocontrole de comportamentos ^17^.

Outro grupo identificado diz respeito às famílias que aplicam consequências para os comportamentos de crianças e adolescentes sem recurso à violência física, com restrição de direitos de circulação ou de usufruto de determinados bens materiais. Aqui, há uma a semelhança com os tipos autoritativos de parentalidade ^16^ ^,^ ^17^. Os dados aqui coligidos não permitem afirmar se os laços familiares são marcados por vínculos sociais e emocionais fortes, características desse tipo de estilo parental ^20^, mas a autoridade aí é exercida de forma democrática, justa, neutra, transparente e respeitosa. Além disso, é menos provável que nesses contextos a violência intrafamiliar esteja presente.

Finalmente, o terceiro grupo foi aquele que apresentou prevalência de vitimizações em casa e o exercício de castigo corporal por parte dos pais. Nesse sentido, assemelha-se à parentalidade autoritária ^16^ ^,^ ^17^, porque os pais ou responsáveis dessas crianças e adolescentes exercem um tipo de autoridade que privilegia o controle sobre o comportamento por meio de punições duras.

A importância deste resultado é a indicação de que os castigos físicos não são uma questão contingencial, mas uma prática comum em grupos familiares em que outras formas de violência intrafamiliar se fazem presentes. Esses dados são preocupantes, pois indicam que essa violência não se manifesta apenas quando as autoridades familiares sentem a necessidade de reafirmar seu poder por meio de punições físicas, mas, sim, porque já está presente de forma mais ampla nas relações interpessoais.

Convém ponderar que, observando a porção do gráfico relativa a este grupo, há uma dispersão relativamente maior dos dados neste *cluster*. O grupo é, portanto, mais heterogêneo que os demais em relação às variáveis consideradas no estudo. Isso exige certa parcimônia ao tirar conclusões sobre os impactos desses resultados. A presença de castigos físicos está associada a outras formas de violência intrafamiliar, mas isso não significa que o castigo corporal só aconteça em lares marcados pela violência intrafamiliar constante. Os resultados sinalizam a importância de considerar as punições corporais como indicativo de violações mais graves em ambiente familiar, ao mesmo tempo que exige atenção e cuidado na identificação desse tipo de maus-tratos.

Notaram-se ainda diferenças significativas entre os grupos com relação a variáveis socioeconômicas fundamentais. Os adolescentes de classe média e alta são aqueles cujas autoridades familiares mais provavelmente abrem mão do castigo físico como forma de imposição de sua autoridade e são os que menos convivem com a violência interpessoal. Ao passo que prevalecem entre as famílias menos privilegiadas modelos de poder mais rígidos, que empregam a força física com maior frequência, especialmente entre os indivíduos que vivem em domicílios com renda mensal menor que dois salários mínimos. Estudos com amostras nacionais já demonstraram a prevalência de violência intrafamiliar entre setores de baixa renda, apontando que a maior escolaridade das mães figura como um fator de proteção ^9^. Nesse sentido, os resultados aqui observados podem refletir um acesso desigual das famílias às informações sobre dos direitos das crianças e adolescentes.

A violência é um fenômeno complexo, de origem difusa e multicausal, e assume, especialmente no Brasil, caráter estrutural e histórico ^26^. Diversas pesquisas apontaram a violência nas relações interpessoais como um padrão de resolução de conflitos que persiste nas relações comunitárias ^27^ ^,^ ^28^ ^,^ ^29^. Desse modo, um aspecto relevante é o da reprodução via socialização das práticas educacionais punitivas, modos de agir que são repassados de geração a geração ^30^.

Para compreender a fundo e atuar sobre esse determinante social da violência, seria necessária uma abordagem sistêmica que lançasse luz sobre os contextos em que a violência é colocada em prática pelos atores sociais ^31^. Nesse sentido, não basta elencar variáveis que formam as condições de fundo do fenômeno, porque um indivíduo vivência a violência em determinadas circunstâncias, em momentos particulares da rotina diária, em situações de interação social específicas ^32^. Compreender a violência enquanto um fenômeno da ordem da interação interpessoal implica considerar como são gerados no ambiente familiar os conflitos que serão resolvidos a base de agressões físicas ^29^. Trata-se, portanto, de investigar como as condições socioeconômicas geram tensão e estresse no ambiente doméstico.

Por sua natureza quantitativa, e por serem oriundos de um survey que não foi inicialmente desenhado para cobrir o fenômeno nesse grau de detalhamento, os dados utilizados nesta análise não permitem uma investigação aprofundada dos contextos e das interações em que essas violências são reproduzidas. Não obstante essa limitação, considerando uma abordagem sistêmica ou interacionista dos conflitos familiares, pode-se elencar algumas hipóteses que expliquem como as condições socioeconômicas atuam na produção de conflitos familiares que resultam em punições corporais e violências intrafamiliares.

Em primeiro lugar, destaca-se que os provimentos mensais inferiores a um salário mínimo sequer conseguem arcar com os produtos da cesta básica alimentar no Brasil ^33^. Assim, os adultos não podem arcar com os custos de apoio profissional para os cuidados com as crianças, tendo de recorrer a redes informais e instáveis de apoio, quando não se veem obrigados, na ausência de tal rede, a deixar as crianças em casa sem supervisão. Essas condições de reprodução social podem aumentar a exposição das crianças às diversas formas de violência urbana no bairro, bem como expô-las a outros comportamentos de risco, situações que, por sua vez, implicarão maior conflitualidade no ambiente doméstico.

Some-se a isso o fato de que nessas famílias frequentemente há apenas um adulto responsável por mais de uma criança. Com provimentos mensais que são insuficientes para as necessidades familiares básicas, sem condições financeiras para os cuidados de supervisão diária, e muitas vezes tendo de assumir sozinhas os cuidados e a educação das suas crianças e adolescentes, as pessoas adultas se veem sob constante pressão e enredadas em um ambiente estressor e reprodutor de conflitos.

Este panorama ressalta a importância de considerar os contextos familiares na formulação de políticas públicas. É necessário criar estratégias que melhorem o acesso a serviços, como redes de apoio social, de modo a aliviar a pressão sobre os pais, principalmente em famílias de baixa renda. Além disso, as políticas públicas devem considerar o peso dos aspectos culturais que normalizam os castigos físicos e das violências intrafamiliares. Isso pode ser feito por meio de programas que promovam habilidades de resolução de conflitos, garantido o acesso a informações sobre os direitos da criança e do adolescente. Nesse sentido, é bem-vinda a promulgação da *Lei nº 14.826/2024* ^34^, que institui a parentalidade positiva e o direito ao brincar como estratégias de prevenção à violência contra crianças.

Mas, em se tratando de políticas de prevenção à violência, é fundamental recordar que, não obstante os esforços legislativos, em termos orçamentários, os planos de enfrentamento à violência ainda carecem de transparência, de dados detalhados, de designação de rubricas e de programas interministeriais que permitam avaliar e monitorar as ações e os investimentos necessários ^35^.

Para a prática cotidiana dos agentes em contato com crianças e adolescentes em situação de violência, espera-se que este artigo contribua para uma compreensão mais abrangente dos desafios enfrentados na prevenção e no enfrentamento dos maus-tratos infantis, oferecendo insights valiosos sobre como lidar com essa questão de forma eficaz e sensível às complexidades das relações familiares e socioeconômicas.

Em sua prática diária, profissionais de saúde podem se deparar com jovens que são (ou foram) vítimas de maus-tratos. Como aponta o documento *Cómo Responder al Maltrato Infantil: Manual Clínico para Profesionales de la Salud*, elaborado pela OMS ^1^, esses profissionais desempenham um papel crucial no reconhecimento e resposta a ações ou omissões que causam danos por vezes irreparáveis.

No estudo realizado por Bannwart & Brino ^36^ abordando médicos pediatras e a notificação de casos de maus-tratos contra crianças e adolescentes, foi observada uma significativa dificuldade em registrar tais casos, motivada por várias razões. Entre elas estão a confiança na dinâmica familiar, as dificuldades na identificação de abuso emocional ou negligência e as preocupações com possíveis consequências negativas da notificação, como falta de mudança e potenciais desvantagens, incluindo o envolvimento com o poder judiciário e possíveis retaliações por parte das famílias afetadas. Apesar de estarem cientes da obrigação de relatar suspeita de maus-tratos (conforme previsto nos artigos 13 e 245 do *Estatuto da Criança e do Adolescente* ^37^), a falta de conhecimento sobre o assunto também se mostra um inibidor da notificação, possivelmente devido a uma desconexão com o contexto social ou à falta de sensibilidade em relação aos impactos da violência intrafamiliar.

Durante exames de rotina, atendimentos de emergência ou consultas gerais, essas crianças ou adolescentes podem chamar a atenção dos profissionais de saúde, seja por problemas de saúde relacionados ou não aos maus-tratos, ou por outras condições médicas e queixas. Assim, quando um profissional de saúde relata um caso de maus-tratos, inicia-se um processo crucial para interromper comportamentos violentos, reconhecendo que tanto as famílias quanto as crianças necessitam.

Os maus-tratos podem não ser autoevidentes, e as vítimas, que são crianças e adolescentes, podem hesitar em compartilhar suas experiências de maus-tratos por diversos motivos (vergonha, culpa ou estigma), além de sequer reconhecerem suas experiências como sendo violentas e temerem represália ^1^. Mesmo que não deixem marcas visíveis, os maus tratos são vestígios de relações familiares marcadas por autoritarismo e violência e, por isso, devem ser investigados com seriedade. Por isso, para notificar os casos de violência, os profissionais precisam saber o que é considerado violência, e a rede de atendimento não conta com pessoas formados para reconhecer as violências. Os resultados aqui coletados recomendam atenção e investigação detalhada, porque o que a sociedade hoje entende como sendo uma mera “palmada” educativa pode revelar riscos mais sérios à saúde e ao bem-estar de crianças e adolescentes.
